# Identification of Putative SNP Markers Associated with Resistance to Egyptian Loose Smut Race(s) in Spring Barley

**DOI:** 10.3390/genes13061075

**Published:** 2022-06-16

**Authors:** Kamal A. M. Abo-Elyousr, Amira M. I. Mourad, P. Stephen Baenziger, Abdelaal H. A. Shehata, Peter E. Eckstein, Aaron D. Beattie, Ahmed Sallam

**Affiliations:** 1 Department of Plant Pathology, Faculty of Agriculture, Assiut University, Assiut 71526, Egypt; elyousr@aun.edu.eg (K.A.M.A.-E.); abdelaal.hamaam@agr.aun.edu.eg (A.H.A.S.); 2 Resources Genetics and Reproduction, Department Genebank, Leibniz Institute of Plant Genetics and Crop Plant Research (IPK), Corrensstr. 3, OT Gatersleben, D-06466 Stadt Seeland, Germany; mourad@ipk-gatersleben.de; 3 Department of Agronomy, Faculty of Agriculture, Assiut University, Assiut 71526, Egypt; 4 Department of Agronomy & Horticulture, University of Nebraska-Lincoln, Lincoln, NE 68583-0915, USA; pbaenziger1@unl.edu; 5 Department of Plant Sciences, University of Saskatchewan, Saskatoon, SK S7N 5A8, Canada; peter.eckstein@usask.ca (P.E.E.); aaron.beattie@usask.ca (A.D.B.); 6 Department of Genetics, Faculty of Agriculture, Assiut University, Assiut 71526, Egypt

**Keywords:** *Hordeum vulgare* L., *Ustilago nuda*, genetic analysis, single-nucleotide polymorphism, candidate genes

## Abstract

Loose smut (LS) disease is a serious problem that affects barley yield. Breeding of resistant cultivars and identifying new genes controlling LS has received very little attention. Therefore, it is important to understand the genetic basis of LS control in order to genetically improve LS resistance. To address this challenge, a set of 57 highly diverse barley genotypes were inoculated with Egyptian loose smut race(s) and the infected seeds/plants were evaluated in two growing seasons. Loose smut resistance (%) was scored on each genotype. High genetic variation was found among all tested genotypes indicating considerable differences in LS resistance that can be used for breeding. The broad-sense heritability (H^2^) of LS (0.95) was found. Moreover, genotyping-by-sequencing (GBS) was performed on all genotypes and generated in 16,966 SNP markers which were used for genetic association analysis using single-marker analysis. The analysis identified 27 significant SNPs distributed across all seven chromosomes that were associated with LS resistance. One SNP (S6_17854595) was located within the *HORVU6Hr1G010050* gene model that encodes a protein kinase domain-containing protein (similar to the *Un8* LS resistance gene, which contains two kinase domains). A TaqMan marker (0751D06 F6/R6) for the *Un8* gene was tested in the diverse collection. The results indicated that none of the Egyptian genotypes had the *Un8* gene. The result of this study provided new information on the genetic control of LS resistance. Moreover, good resistance genotypes were identified and can be used for breeding cultivars with improved resistance to Egyptian LS.

## 1. Introduction

Barley (*Hordeum vulgare* L.) is one of the most important cereal grains grown in temperate climates. It has been used for animal feed (as a grain or as fodder) and human nutrition (as a component of various foods). During its life cycle, barley suffers from many biotic and abiotic stresses which affect its yield. Loose smut (LS) (caused by the basidiomycete pathogen *Ustilago nuda* (Jens.) Rostr. (*U. nuda*)) is one of these important diseases. This disease has been reported in 1.3–11.7% of fields in Egypt [1], 50–70% of fields in Western Canada [2], and it is also common in the Great Plains of the United States [3]. Certified barley seed is highly recommended to be treated with fungicide to prevent this disease. During grain development, the smutted (diseased) heads disperse spores by wind or rain to healthy heads. After harvest, the infected seeds with LS appear normal. However, in the next season, after germination, the fungus will infect and grow within the plant and replace the head with spores at the flowering time [4]. A reduction in yield is directly associated with the percentage of infected tillers. Climate change is expected to increase the pathogen spread by increasing temperatures, changing precipitation patterns, changing extreme weather events, and reducing water availability [5]. Therefore, the chances of agriculture crops encountering both biotic and/or abiotic stresses are expected to be more frequent than in the past. This change will result in a significant deterioration in staple crops such as barley and wheat (*Triticum aestivum* L.). 

Loose smut is a particular problem for organic and low-input agriculture as fungicidal seed treatments are not applied. Therefore, breeding resistant barley cultivars for LS is the main approach to reducing the devastating effects of LS in these cropping systems. It is equally important that the new cultivars combine both good agronomic performance and LS resistance. [6,7] reported *Un* as the first resistance gene in the cv. ‘Trebi’ in the 1940s. Thereafter, 15 resistance genes were found to be associated with true loose smut resistance [8,9,10,11,12]. Of the 15 genes, *Un8* showed durable resistance against all true loose smut isolates that were observed in Western Canada [13].

Traditional plant breeding programs have been reported as a very successful tool for developing cultivars showing resistance to diseases. However, plant breeding is a time-consuming and labor-expensive or intensive process as it largely depends on field evaluation and selection [14]. Molecular marker-assisted selection (MAS) for true loose smut resistance is an alternative approach and can be useful in accelerating breeding for resistance, as well as improving agronomic performance. Significant progress has been made in identifying molecular markers that show a strong linkage with loose smut-resistant genes like *Un8* [15]. SSR and RFLP markers were used to initially tag *Un8* [3,16]. However, when single nucleotide polymorphism (SNP) maps became available for barley, it was feasible to develop additional markers for *Un8*. Recently, Zang [17] identified two flanking SNP markers located very close to *Un8*. Since SNP markers provide valuable and more reliable tagging than other types of DNA markers, SNP markers close to *Un8* can be used for breeding resistance to true loose smut. However, SNP markers associated with the other 15 barley loose smut resistance genes are lacking. To date, there is no information on DNA markers associated with resistance against 98 Egyptian loose smut races.

More information is needed to understand the genetic control of LS resistance in barley globally. In our study, barley resistance to LS under Egyptian conditions was investigated.

The objectives of this study were to (1) examine the genetic variation among Egyptian and spring barley genotypes in their resistance to Egyptian LS races, (2) identify putative SNP markers and genomic regions associated with LS resistance to be used in marker-assisted selection for loose smut resistance in barley, and (3) select the most resistant genotypes which could be used in breeding programs to genetically improve LS resistance for Egyptian barley production.

## 2. Materials and Methods

### 2.1. Plant Materials

A total of 62 highly diverse spring barley genotypes were included in this study. The seeds of 60 genotypes were obtained from the Agriculture Research Center (ARC), Egypt, and two from the United States Department of Agriculture (USDA), USA. The complete list of the genotypes and the pedigree is represented in Supplementary Table S1. The tested genotypes included 2-rows, 6-rows, and naked and hulled barley genotypes. The genotypes were divided based on their population structure into two subpopulations based on their population structure, genetic diversity, and the possibility of using them to improve different traits relevant to Egyptian growing conditions [18].

### 2.2. Evaluation of LS Resistance

The genotypes were inoculated with the Egyptian race(s) of LS in the greenhouse of the Plant Pathology Department, Assiut University, Egypt, in 2017/2018. An inoculation liquid was prepared by mixing 1 gm of *U. nuda* spores collected from the Egyptian fields in the previous years with 1 L of distilled water [19]. For each tested genotype, 6–8 heads were inoculated using a 3 mL syringe. After harvest, inoculated spikes from each genotype were collected. The LS resistance was evaluated in the 2018/2019 and 2019/2020 growing seasons at the heading stage, according to Eckstein et al. [16]. The infected seeds were planted in the greenhouse in a randomized complete block design (RCBD) with three replications. The infected and health spikes for each genotype were counted and the resistance was calculated as a percentage of the infected spike divided by the total number of spikes. The level of resistance was determined based on [20] using a scale as follows: percentage of infected heads 0% = immune genotype, 0.1–5% (highly resistant) genotype, 5.1–10% (resistant), 10.1–20% (moderately resistant), 20.1–30% (moderately susceptible), 30.1–50% (susceptible), and 50.1–100% (highly susceptible).

### 2.3. Statistical Analysis of LS Resistance

An analysis of variance (ANOVA) of LS resistance was conducted using R software [21] Data from both years were combined and the following model was used:

*Y_ijk_* = *µ* + *g_i_* + *r_j_* + *y_k_+ gy_ik_* + *e*_*ijk*_,

where *Y_ijk_* is the disease reaction of genotype *i* in replication *j* planted in year *k*, *µ* is the general mean; *g_i_*, *r_j_*, and *y_k_* are the main effects of genotypes (fixed effects), replications, and years (random effects), respectively. *gy_ik_* is genotype × year interaction and; *e_ijk_* is the error. The broad-sense heritability (H^2^) was calculated as follows:

$$H^{2}=\sigma_{G}^{2}/\left(\sigma_{G}^{2}+\frac{\sigma_{R}^{2}}{ry}\right)$$

where $\sigma_{G}^{2}$ and $\sigma_{R}^{2}$ are the variance of the lines and the residuals, *r* is the number of replicates within the experiment, and *y* is the number of years.

### 2.4. DNA Extraction and Genotyping-by-Sequencing (GBS)

DNA was extracted from all the 60 genotypes by collecting 4–5 leaves from five-day-old barley seedlings. The extraction protocol was conducted as described in Mourad et al. [22]. DNA concentration was measured using spectrophotometry (Gen5TM microplate reader and image software with Take3TM micro-volume plates (BioTek, Winooski, VT, USA). DNA of each genotype was digested using two restriction enzymes, *PstI* and *MspI,* for genotyping-by-sequencing as described in [23]. Single nucleotide polymorphism (SNP) calling used the TASSEL 5.0 v2 GBS pipeline [24]. Identification of SNP markers, their physical position, and localization was carried out using the Barley cv. MorexV2 genome assembly. A set of 25,700 SNPs was obtained from the GBS data that was filtered for minor allele frequency (MAF >0.05), maximum missing sites per SNP <20%, and maximum missing sites per genotype <20%. Heterozygous loci were then marked as missing to obtain better estimates of marker effects (Peter Bradbury, personal communication) and the filtration was repeated based on the previous criteria.

### 2.5. Single Marker Analysis of LS Resistance and Linkage Disequilibrium

The phenotypic data (loose smut resistance, %), as well as the available SNP markers, were used to perform a single-marker analysis (SMA) to identify SNP markers significantly associated with resistance. Single marker analysis was performed using PowerMarker software V 3.25 [25] using the following model:

*Y* = *µ* + *f* (marker) + error,

where *Y* is equal to the trait value, *µ* is equal to the population mean, and *f* (marker) is a function of the significant markers. The phenotypic variation explained by each significant SNP marker was estimated using TASSEL 5.0 software [24].

For significant SNP markers located on the same chromosome, linkage disequilibrium (LD, *r*^2^) was calculated using TASSEL 5.0 and visualized as a heatmap using “Ldheatmap” R package [26]. The genetic distance among immune genotypes was calculated based on their resistance alleles using a simple matching approach. The genetic distance calculation was performed using the ade4 R package [27].

### 2.6. Gene Models Underlying Significant SNPs and Their Validation

To further confirm the SMA results, we investigated if any of the significant SNPs were located within gene models identified in the reference genome assembly published by the International Barley Genome Consortium (IBSC V.2) available on Ensemblplants (http://plants.ensembl.org/Hordeum_vulgare/Info/Index, accessed on 12 June 2022). The functional annotation of the identified gene models was retrieved from the genome annotation provided by IBSC_V2 and examined for their association with disease resistance. The expression of candidate genes was investigated from the Expression Atlas database (https://www.ebi.ac.uk/gxa/home, accessed on 12 June 2022).

### 2.7. Screening of the Genotypes for the Presence of the Un8 LS Resistance Gene

The 0751D06 F6/R6 TaqMan^®^ assay, located within the *Un8* gene, was developed by Zang et al. [17] to screen for the presence of Un8 resistance or susceptible alleles. The 60 barley genotypes were genotyped using this marker at the Department of Plant Sciences, the University of Saskatchewan. TaqMan^®^ SNP genotyping was performed with the ABI StepOnePlus™ Real-Time [17]. Five genotypes were used as checks for the *Un8* gene. TR12135 and AC Metcalfe were characterized as resistant to loose smut and carried the *Un*8 gene, while TR14150, Harrington and Xena were characterized as susceptible and lack the *Un8* gene [17]. The seeds of checks were obtained from the Department of Plant Sciences, University of Saskatchewan, Saskatoon, SK, Canada.

## 3. Results

### 3.1. Phenotypic Variation in Loose Smut Resistance in the Tested Genotypes

The analysis of variance for loose smut resistance is presented in Table 1. The ANOVA revealed highly significant differences among the genotypes for loose smut resistance. No significant differences were found within the main effect of the year or within the genotypes × replications and genotypes × years interaction effects. Broad-sense heritability was high based on the average of the two years (H^2^_B_ = 0.95). 

**Figure 1:**
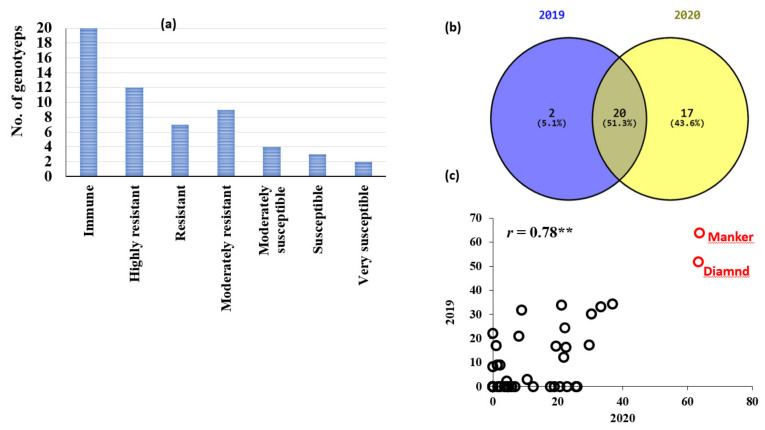
(**a**) Distribution of loose smut reaction among the 60 genotypes comprising the Egyptian spring barley collection, (**b**) Venn diagram illustrating the identification of immune genotypes in 2019 and 2020, (**c**) Correlation of loose smut reactions measured in 2019 and 2020 among the genotypes demonstrating an immune reaction in at least one year. ** *p* < 0.01.

**Figure 2:**
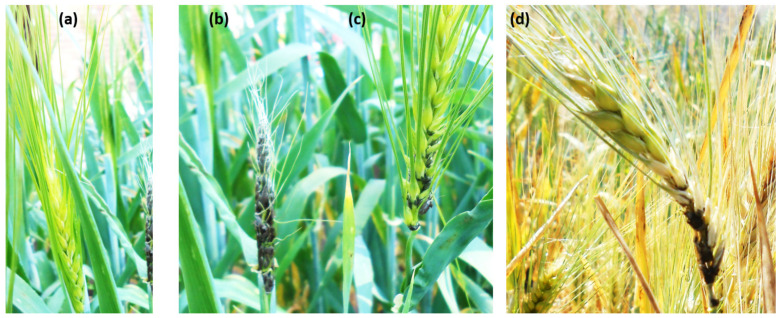
The effect of the Egyptian loose smut race on barley spikes (**a**) health spikes (**b**) susceptible spikes, (**c**,**d**) different degrees from few partially infected spikes found in some resistant genotypes.

The 57 genotypes displayed different degrees of infected heads ranging from 0 to 64% based on the average of both years. All genotypes could be classified into six groups: immune (20 genotypes), highly resistant (12 genotypes), resistant (seven genotypes), moderately resistant (nine genotypes), moderately susceptible (four genotypes), susceptible (three genotypes), and highly susceptible (two genotypes) (Figure 1a). A set of 22 genotypes were immune in the 2019 experiment. Whereas in the 2020 trial, this number increased to 37 genotypes with 0% infected heads (Figure 1c). Twenty genotypes had no infected spikes in the two years. A positive significant correlation was found between the two years (*r =* 0.78 **) for LS resistance (Figure 1c). The highly resistant genotypes showed only healthy spikes (Figure 2a), the susceptible genotypes had fully infected spikes (Figure 2b), while the moderately resistant genotypes showed partially infected spikes (Figure 2c,d). 

### 3.2. Single-Marker Analysis of Loose Smut Resistance against the Egyptian Race and Linkage Disequilibrium between the Significant SNPs

**Figure 3:**
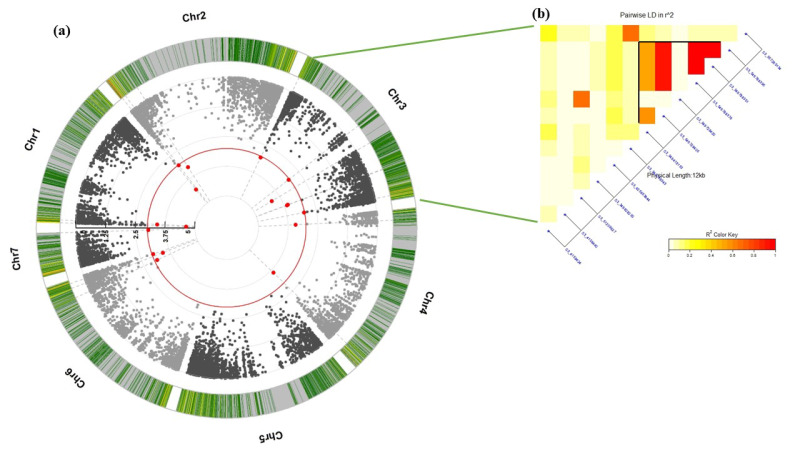
(**a**) Manhattan plot showing the significant SNPs (red circles) associated with LS resistance and their locations on barley chromosomes. The red line indicated the significant threshold at 0.001, (**b**) LD (*r^2^*) among significant SNPs located on chr 3H.

Genotyping-by-sequencing generated a set of 25,700 SNPs. After filtration, the number decreased to 16,966 SNPs used for SMA and further genetic analysis. Due to the absence of the genotypes x year interaction, average LS values obtained from both years were used in the SMA analysis. The SMA identified 27 SNP markers significantly associated with the resistance (*p*-value < 0.001) (Table 2, Figure 3a). These significant SNPs were distributed among the seven chromosomes of the barley genome, with the highest number on chromosome 3H (13 SNPs) and the lowest number on chromosomes 5H and 6H (one SNP each). The phenotypic variation of the significant SNPs (R^2^) ranged from 0.55 % for SNP S3_4158424 (3H) to 47.70% for SNP S2_6903688 (2H). The four significant SNPs identified on chromosome 2H were found to have the highest R^2^, with values ranging from 16.12% to 47.70%. The target alleles of the significant SNPs decrease the symptoms of loose smut in the genotypes with a percentage ranging from 1.71% for SNP S3_4158424 to 23.57% for SNPs S3_546788379, S3_546788391, and S3_546788395.

LD (*r^2^*) between each pair of significant SNPs located on the same chromosome was calculated. The highest number of high LD genomic regions was found on chromosome 3H among the 13 significant SNPs (Figure 3b). Complete LD was found among S3_546788395, S3_546788391, and S3_546788379. The entire LD block was divided by SNP S3_546728420 due to missing data associated with this SNP. In addition, high LD was found between S3_546728420 and S3_51209627, and between S3_546788379 and S3_543138543. Two SNPs located on chromosome 4H were incomplete LD (Supplementary Figure S1c). No significant LD was found between the significant SNPs located on chromosome 2H nor 7H (Supplementary Figure S1a,b).

### 3.3. Genes Underlying Significant SNPs and Their Validation

**Figure 4:**
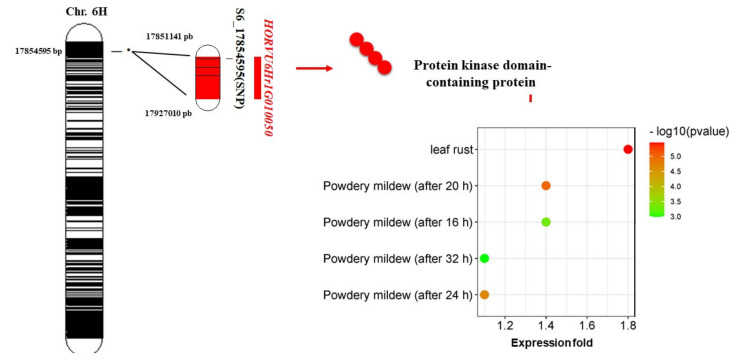
Position and candidate gene with its annotation for S6_17854595 SNP associated with loose smut resistance.

To further understand the genetic association between the significant SNP markers and loose smut resistance in the tested genotypes, gene models harboring these SNPs and their functional annotation were investigated (Table 3). Gene models that were located within or near significant SNPs were determined. Eleven of the SNPs were not located near or within any gene models. The HORVU3Hr1G002060 gene model was found to be 455 and 439 bp from two SNPs, S3_4158424 and S3_4158440, respectively, located on chromosome 3H. This gene model encodes a protein belonging to the O-acyltransferase (WSD1-like) family. The S6_17854595 SNP located on chromosome 6H was found to be located within a gene model *HORVU6Hr1G010050* which encodes a protein kinase domain-containing protein (Figure 4).

### 3.4. Genotyping with Un-8 Taqman Marker

The presence or absence of the *Un8* gene was tested using the 0751D06 F6/R6 TaqMan^®^ assay. To confirm the genotyping results, two positive checks and three negative checks for the *Un8* gene were used along with the 62 genotypes. The results revealed that none of the genotypes used in this study possessed the *Un8* gene (Supplementary Table S1).

### 3.5. Selection of Superior Loose Smut Resistance Genotypes

**Figure 5:**
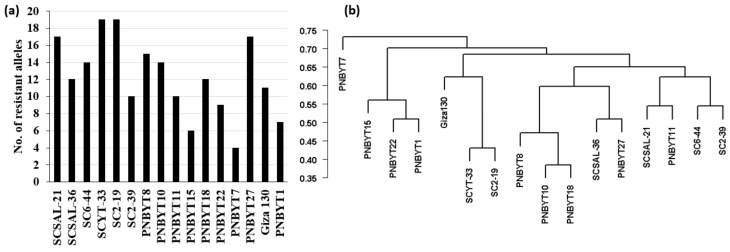
(**a**) number of resistant alleles in the most resistant genotypes and (**b**) genetic distance and dendrogram analyses among the most resistant genotypes based on the presence of resistance alleles.

Out of the 20 immune genotypes (Supplementary Table S1), only 16 genotypes had available sequence data after filtration criteria. To genetically confirm the superior resistance in these 16 highly resistant genotypes, the number of targeted alleles (favorable alleles) of the 27-significant SNP markers was investigated in each resistant genotype (Figure 5a, Supplementary Table S2). This number ranged from 4 to 19 SNP markers. Two genotypes, SC2-19 and SCYT-33 contained the highest number of targeted SNPs. The PNBYT7 cultivar was found to be carrying the lowest number of targeted SNP alleles (four alleles). Furthermore, the presence of the targeted alleles of the two SNP markers with the highest allele effect (S2_6903688 and S2_71163470) was investigated in the genome of these resistant genotypes. Most of the resistant genotypes (12 genotypes) were found to contain the targeted allele of S2_71163470 SNP. However, only five of these genotypes carried the targeted allele of S2_6903688 SNP.

To further understand the possible improvement of loose smut resistance in spring barley using these genotypes, the genetic distance between each pair of resistant genotypes was calculated based on the significant SNPs (Supplementary Table S3) and presented as a dendrogram cluster (Figure 5b). Based on the dendrogram cluster, the two genotypes with the highest number of targeted SNPs, “SCYT-33 and SC2-19”, were located in the same cluster. These two genotypes differed in six alleles. The PNBYT7 genotype was located in a separate cluster from the remaining 15 genotypes.

## 4. Discussion

As loose smut is one of the major diseases that affect the production of barley globally, it is very important to understand the genetic basis of resistance. No previous studies related to resistance against Egyptian LS race(s) has been conducted. Up to our knowledge, our study could be considered as the first report on the genetics of LS resistance against Egyptian LS. Understanding such an important point will accelerate the production of barley, not only in Egypt but also in the different parts of the world where LS disease occurs, and organic agriculture is practiced. In the current study, the presence of highly susceptible genotypes (with the percentage of infected heads >50%) in both years indicated the successfulness of the artificial infection and the validity of the results.

### 4.1. Genetic Variation in Loose Smut Resistance

The presence of highly significant differences among the tested genotypes in their response to loose smut indicated our artificial inoculation and phenotypic assay were successful. The resistant genotypes would be very useful in improving resistance to this disease when grown in Egyptian environments. The high correlation in disease reaction response of the tested genotypes and the absence of G × Y interaction to LS in the two years indicated that consistent response to LS. The high degree of broad-sense heritability (H^2^_B_ = 0.95) also indicated that the phenotypic variation of LS resistance in the studied materials is stable and mainly due to genotypic variation. As a result, improving LS resistance in spring barley could be possible due to the successful selection of highly resistant genotypes, which will be very useful, especially for low input and organic fields where seed treatment with fungicides is not practiced. The different degrees of the partially infected spikes (Figure 1a) may be indicated by the presence of different loose smut races, as those genotypes presented different infection types on their spikes. In the partially infected spikes, the basal parts of the infected spike are smutted, with the distal parts producing normal grains [28].

Twenty of the evaluated genotypes were found to be immune to LS in both years. Most of these genotypes were 6-rows (12 genotypes) or naked (14 genotypes), with six of them having both traits (naked 6-row type). It was known that 6-row barley genotypes are highly fertile and high-yielding due to the formation of kernels from all the spikelet’s flowers. Naked barley genotypes were also preferred as they are linked directly to dietary food uses [29]. The presence of LS resistance in naked 6-row barley will allow the improvement of this important barley type for Egyptian barley production. Diamond from Canada and Wocus 71 (CIho 15554) from the USA had the highest number of infected spikes and were considered highly susceptible to LS. Therefore, these two genotypes can be used as susceptible checks against the Egyptian race of LS in future loose smut experiments in barley to validate and calibrate the successful inoculation with the fungus.

### 4.2. Genetic Analysis of LS Resistance

To fully utilize the resistant genotypes identified in this study, it is very important to understand the genetic control of LS. In this study, the genotyping was undertaken using 16,966 SNP markers which were highly polymorphic and distributed across all genotypes.

The genetic association can be carried out using different kinds of analysis, such as genome-wide association study (GWAS) and single-marker analysis (SMA). In this study, the 60 genotypes used were not appropriate for GWAS studies, which require at least 100 genotypes to be precise [30]. However, SMA can be carried out using any number of genotypes, hence, it would be appropriate for this study [31,32]. Using the same set of genotypes and SNP, the single-marker analysis was successful to identify genomic regions associated with other target traits in barley, such as drought tolerance [33] and heat tolerance [34].

Single-marker analysis identified a set of 27 SNP markers associated with LS resistance in the studied genotype materials based on the average of both years data. The chromosomal location of the identified SNPs indicated the presence of multiple genomic regions controlling resistance (Figure 3). Based on the R^2^ of the significant SNPs, all SNPs with R^2^ less than 10% were considered as marking minor effects (chromosomes 6H and 7H). The SNPs located on chromosomes 1H, 2H, 4H, and 5H could be considered as marking major effect loci (R^2^ > 10%) [35,36,37]. Two regions on chromosome 3H, as determined by low LD between two distinct groups of SNPs, were associated with LS resistance, with the majority considered as marking a major QTL. The different degrees of LD between each pair of significant SNPs on the same chromosome indicated the presence of multiple QTLs/genes. High LD SNPs were found on the 3H chromosome and indicated that these genomic regions tend to co-inherited together. As a result, we can conclude the presence of multiple genes/QTLs controlling the resistance against the Egyptian race of LS. The resistance alleles identified by these SNPs were associated with a decrease in loose smut symptoms from 1.7 to 23.9%. Chromosome 3H had a high LD genomic region of three SNPs (S3_546788379, S3_546788391, and S3_546788395) that decreased loose smut resistance by 23.92%. Therefore, these markers could be useful for breeding loose smut resistance in barley.

To further understand the genetic association between the identified significant SNPs and LS resistance, gene models harboring or located near significant SNPs were investigated. The annotation revealed 15 gene models. Out of the 15 gene models, three were identified to be located very near the significant SNPs on chromosome 1H (one gene model) and 3H (two gene models). The gene model identified on chromosome 1H was functionally annotated as fatty acyl-CoA reductase (FAR), an enzyme that is responsible for alcohol and fatty acid formation. Fatty acids were reported to be a very important regulator of plant defense against different microbes [38,39], while the functional annotation of the two gene models on chromosome 3H encodes O-acyltransferase (WSD1-like) family. The *HORVU3Hr1G002060* gene model was located near S3_4158424 and S3_4158440. These two SNPs were located before the gene model at a distance of 439 and 455 bp, respectively. O-acyltransferase (WSD1-like) family plays an important role in synthesizing different types of waxes [40]. These wax components are transported from the endoplasmic reticulum to the plasma membrane via the Golgi and consequently transported out of the plant cell to the cuticle layer. The plant cuticle consists of a cutin scaffold covered with cuticular waxes [41,42] that were reported to play a vital role in plant defense against pathogens by acting as a plant’s physical barrier, regulating the plant–pathogen interactions [9,43].

Of all significant SNPs identified, one SNP on chromosome 6H is located within a gene model of *HORVU3Hr1G002060*. This gene encodes a protein kinase domain-containing protein. Protein kinase domains can be divided into two phosphorylating groups, phosphorylating either serine/threonine or tyrosine residues on target proteins [44]. Serine/threonine protein kinases have been associated with plant disease resistance pathways [41]. Such proteins have previously been associated with resistance to powdery mildew (*Blumeria graminis* f. sp. *tritici*) in wheat [41], stem rust resistance in barley [42], bacterial blight disease resistance in rice [12], and stripe rust resistance in wheat [43]. HORVU6Hr1G010050 was found to be highly expressed 1.8-fold under stem rust resistance compared to the control (Figure 4, Supplementary Table S4). Moreover, it also had a higher expression under powdery mildew than under control. Therefore, the S6_17854595 located on the 6H chromosome could be very useful to target the *HORVU6Hr1G010050* gene. The result of gene annotation of this SNP supports the power of genetic association performed in this study in detecting putative SNP associated with loose smut resistance. A KASP marker targeting this SNP can be designed and used for validation in other genetic backgrounds before using it for marker-assisted selection to improve resistance to LS in barley. The expression of the other candidate gene models did not associate with disease resistance.

As mentioned previously, among the 15 different LS resistance genes reported, *Un1* to *Un15*, only the *Un8* gene has a defined chromosomal location [17]. Due to the lack of enough available information about the LS resistance genes in barley, it is very difficult to determine if the known resistance genes might exist in this current studied material. Hence, more studies should be conducted to provide a deep understanding of the genetic control of LS resistance under Egyptian conditions.

### 4.3. Genotyping of Un8 LS Resistant Gene

The *Un8* resistant gene was reported as an important gene that is very effective against all LS races in Europe and Canada [17,45]. There is no available information about the effectiveness of this gene against Egyptian LS races, therefore, it was important to test the effectiveness of this gene against Egyptian races. The 0751D06 F6/R6 *Un8*-specific marker (A/G) was used to evaluate the 60 Egyptian genotypes, along with two resistant checks (AC Metcalfe and TR12135). All genotypes were found to be non-carriers of the *Un8* gene. The immune genotypes thus appear to carry other genes for LS resistance. Further trials are needed to evaluate the effectiveness of this gene against the Egyptian race, as integrating this gene into the Egyptian barley gene pool could improve resistance to Egyptian LS races.

### 4.4. Selection of Superior Genotypes to LS Resistance

According to the phenotypic and genotypic criteria used in our selection, the best genotypes that can be used for crossing would be SCYT-33 and PNBYT15 for the following reasons; (I) the genetic distance between these two resistant genotypes was 0.88, (II) they were found to have 19 and 6 favorable marker alleles, respectively, and (III) a set of 19 different favorable alleles were found between them and shared three favorable alleles. Therefore, cultivars having 22 favorable alleles may be produced from crossing between these two genotypes. Although SCYT-33 and SC2-19 had the highest number of target alleles, they had the lowest genetic distance (0.43). The number of different alleles between the two genotypes was 9 and they shared 13 favorable alleles and were located in the same subpopulation [18]. Therefore, crossing these two genotypes could not be as effective as crossing between SCYT-33 and PNBYT15. Crossing between two far genetic distance genotypes with as many as target alleles could produce lines with distinct alleles controlling the trait and high combining ability [46]. An additional concern that should be taken into account when using SCYT-33 and SC2-19 as parents in breeding for LS resistance is that both genotypes have hulled grain heads which are not preferred in the food industry. In addition, the lowest number of target alleles markers were found in PNBYT7 (four alleles’ markers). This genotype was found to have a high genetic distance from SCYT-33 and SC2-19, indicating that crossing between these two genotypes and PNBYT7 could improve the resistance in this genotype too. Based on our results, gene pyramiding can be used to produce genotypes with durable LS resistance and could be achieved by crossing between resistance parents.

## 5. Conclusions

The results of this study provide valuable information on the genetic improvement of loose smut resistance in barley, especially in the context of Egyptian barley production. A set of 27 SNPs were reported to be associated with loose smut resistance. Of the 27 SNPs, one SNP located on chromosome 6H could be a promising marker as it is within a gene model encoding a protein kinase previously associated with resistance to other plant pathogens. Moreover, the 0751D06 F6/R6 marker targeting the *Un8* resistance genes demonstrated that this gene was not present in the Egyptian genotypes. Promising resistant genotypes were identified that could be used for future breeding programs to improve loose smut resistance in barley.

## Figures and Tables

**Table 1:** Analysis of variance for loose smut resistance in the 60 genotypes comprising the Egyptian spring barley collection.

Source	d.f	M.S.
Years (Y)	1	13.79
Replications (R)	2	216.30 *
Genotypes (G) GR	56 112	1231.26 ** 51.91
GY	56	58.33
GYR	110	46.53
Heritability	0.95	

* p < 0.05, ** *p* < 0.01.

**Table 2:** Single marker analysis (SMA) for loose smut resistance in the 60 barely genotypes comprising the Egyptian spring barley collection.

SNP ID	Chrom.	*p*-Value	F-Value	R^2^	Target Allele	Allele Effect
S1_523594	1H	0.000652344	13.86	27.256	G/A	−15.54%
S2_6903688	2H	3.37 × 10^−5^	24.62	47.70	C/G	−14.57%
S2_6903698		0.000697591	14.65	35.172	G/C	−15.57%
S2_71163470		0.000146541	18.71	37.643	C/T	−21.92%
S2_481116363		0.000366765	16.12	34.949	A/G	−13.15%
S3_4158424	3H	0.000906049	8.282851873	0.545	C/T	−1.71%
S3_4158440		0.00081791	8.424957364	1.085	C/T	−3.23%
S3_51209627		0.000847857	8.374949083	17.162	T/C	−9.57%
S3_346828235		0.00072923	8.585119232	24.637	C/T	−17.09%
S3_425653844		0.000956134	8.208412451	19.239	T/C	−9.89%
S3_543138543		0.000282644	9.941050754	14.42	G/A	−8.31%
S3_546418133		0.000758585	8.529945709	27.916	T/C	−17.86%
S3_546728405		1.85 × 10^−5^	14.18761801	26.1	G/A	−19.87%
S3_546728420		0.000332253	9.705460245	27.021	A/G	−13.42%
S3_546788379		0.000127966	11.12149677	33.878	T/A	−23.57%
S3_546788391		0.000127966	11.12149677	33.878	A/G	−23.57%
S3_546788395		0.000127966	11.12149677	33.878	C/A	−23.57%
S3_557269134		0.000761867	8.523916211	24.861	T/A	−10.69%
S4_510236504	4H	0.000617005	8.819871045	16.644	A/G	−8.94%
S4_510236509		0.000617005	8.819871045	16.644	A/G	−8.94%
S5_8264909	5H	0.000755518	8.53560527	22.878	A/G	−10.61%
S6_17854595	6H	0.000986781	8.164849355	5.432	A/G	−7.81%
S7_10286228	7H	0.000558655	8.960296029	7.383	G/A	−8.22%
S7_27627925		0.000429506	9.335043364	0.776	T/C	−2.17%
S7_50465300		0.000980333	8.173896443	3.264	A/G	−3.92%
S7_50465325		0.000980333	8.173896443	3.264	A/G	−3.92%
S7_54495415		0.000855639	8.36225696	7.226	C/G	−7.61%

**Table 3:** The genomic location and annotation of gene models harboring SNP markers significantly associated with loose smut resistance in the 60 Egyptian genotypes.

SNP ID	Chrom.	Gene Model	Gene Model Position (bp)	Pos. (Gene Model)	GD (Gene Model)	Gene Annotation
S1_523594	1H	*HORVU1Hr1G000190*	514755-520291	after	3033	Fatty acyl-CoA reductase
S2_6903688	2H	*NA*	NA			NA
S2_6903698						
S2_71163470		*NA*	NA			NA
S2_481116363		*NA*	NA			NA
S3_4158424	3H	*HORVU3Hr1G002060*	4158879-4162702	before	455	O-acyltransferase (WSD1-like) family
S3_4158440		*HORVU3Hr1G002060*	4158879-4162702	before	439	O-acyltransferase (WSD1-like) family protein
S3_51209627		*HORVU3Hr1G019140*	51126683-51134627	after	75000	receptor kinase 1
S3_346828235		*HORVU3Hr1G049410*	346862885-346863988	before	34650	Disease resistance protein RPM1
S3_425653844		*HORVU3Hr1G056930*	425800602-425801141	before	146758	Protein H
S3_543138543		*HORVU3Hr1G071870*	543136922-543138097			Uncharacterized protein
S3_546418133		*NA*	NA			NA
S3_546728405		*NA*	NA			NA
S3_546728420		*NA*	NA			NA
S3_546788379		*NA*	NA			NA
S3_546788391		*NA*	NA			NA
S3_546788395		*NA*	NA			NA
S3_557269134		*HORVU3Hr1G074130*	557216044-557217268	after	51866	Holliday junction DNA helicase
S4_510236504	4H	*HORVU4Hr1G060790*	510209263-510210332	after	26172	unknown function
S4_510236509		*HORVU4Hr1G013970*	510209263-510210332	after	27246	unknown function
S5_8264909	5H	*NA*	NA			NA
S6_17854595	6H	*HORVU6Hr1G010050*	17851141-17927010	within	-	Protein kinase domain-containing protein
S7_10286228	7H	*HORVU7Hr1G007880*	10273667-10273950	after	12278	HAT family dimerisation domain containing protein, expressed
S7_27627925		*HORVU7Hr1G020360*	27617705-27618557	after	9368	undescribed protein
S7_50465300		*HORVU7Hr1G028130*	50500827-50501771	before	35527	NA
S7_50465325		*NA*	NA	NA	NA	NA
S7_54495415		*HORVU7Hr1G029150*	54451633-54453868	after	41547	Ubiquitin-like domain-containing protein

## Data Availability

The sequence data presented in this study are not publicly available due to some ongoing projects on the same plant materials. Other data is presented in the Supplementary Files.

## Supplementary Materials

The following supporting information can be downloaded at: https://www.mdpi.com/article/10.3390/genes13061075/s1, Table S1: Detailed information for the 60 genotypes comprising the Egyptian spring barley collection; Table S2: Presence of favorable alleles in the 16 immune genotypes; Table S3. Genetic distance calculated among the 16 immune genotypes with available sequence data; Table S4. Expression analysis of HORVU6Hr1G010050 gene model under different fungal diseases; Figure S1. Linkage disequilibrium between each pair of significant SNPs located on chromosomes 2H (a), 7H (b), and 4H (c).

## References

[1] Cappers R., Fantone F., Neef R., van Doorn C. (2012). Archaeobotanical Evidence of the Fungus Covered Smut (*Ustilago hordei*) in Jordan and Egypt. Analecta Prashist. Leiden..

[2] Tekauz A. (2003). Diseases of Barley. Diseases of Field Crops in Canada.

[3] Menzies J.G., Steffenson B.J., Kleinhofs A. (2010). A Resistance Gene to Ustilago Nuda in Barley Is Located on Chromosome 3H. Can. J. Plant Pathol..

[4] Wilcoxson R.D., Saari E.E., Hettel G., McNab A. (1996). Bunt and Smut Diseases of Wheat: Concepts and Methods of Disease Management.

[5] Kissoudis C., van de Wiel C., Visser R.G.F., van der Linden G. (2014). Enhancing Crop Resilience to Combined Abiotic and Biotic Stress through the Dissection of Physiological and Molecular Crosstalk. Front. Plant Sci..

[6] Livingston J.E. (1942). Inheritance of Resistance to Ustilago Nuda. Phytopathology.

[7] Robertson D.W., Wiebe G.A., Shands R.G. (1947). A Summary of Linkage Studies in Barley: Supplement II, 1947–1953. Agron. J..

[8] Schaller C.W. (1949). Inheritance of Resistance to Loose Smut, Ustilago Nuda, in Barley. Phytopathology.

[9] Skoropad W., Johnson L. (1952). Inheritance of Resistance to Ustilago Nuda in Barley. Can. J. Bot..

[10] Andrews J. (1956). Inheritance of Reaction to Loose Smut, Ustilago Nuda, and to Stem Rust, Puccinia Graminis Tritici, in Barley. Can. J. Agicultural Sci..

[11] Metcalfe D.R. (2011). Inheritance of Loose Smut Resistance: III. Relationships between the “Russian” and “Jet” Genes for Resistance and Genes in 10 Barley Varieties of Diverse Origin. Can. J. Plant Sci..

[12] Chen S., Wang C., Yang J., Chen B., Wang W., Su J., Feng A., Zeng L., Zhu X. (2020). Identification of the Novel Bacterial Blight Resistance Gene Xa46(t) by Mapping and Expression Analysis of the Rice Mutant H120. Sci. Rep..

[13] Thomas P.L., Menzies J.G. (1997). Cereal Smuts in Manitoba and Saskatchewan, 1989–1995. Can. J. Plant Pathol..

[14] Jiang G.-L. (2013). Plant Marker-Assisted Breeding and Conventional Breeding: Challenges and Perspectives. Adv. Crop Sci. Technol..

[15] Mayer K.F.X., Martis M., Hedley P.E., Simková H., Liu H., Morris J.A., Steuernagel B., Taudien S., Roessner S., Gundlach H. (2011). Unlocking the Barley Genome by Chromosomal and Comparative Genomics. Plant Cell.

[16] Eckstein P.E., Krasichynska N., Voth D., Duncan S., Rossnagel B.G., Scoles G.J. (2002). Development of PCR-Based Markers for a Gene (Un8) Conferring True Loose Smut Resistance in Barley. Can. J. Plant Pathol..

[17] Zang W., Eckstein P.E., Colin M., Voth D., Himmelbach A., Beier S., Stein N., Scoles G.J., Beattie A.D. (2015). Fine Mapping and Identification of a Candidate Gene for the Barley Un8 True Loose Smut Resistance Gene. Theor. Appl. Genet..

[18] Sallam A., Amro A., El-Akhdar A., Dawood M.F.A., Kumamaru T., Baenziger P.S. (2018). Genetic Diversity and Genetic Variation in Morpho-Physiological Traits to Improve Heat Tolerance in Spring Barley. Mol. Biol. Rep..

[19] Mishra R., Tiwari S., Khare M. (1990). Studies on Loose Smut of Wheat. X. Testing of Resistance and Susceptibility of Wheat Varieties to *Ustilago tritici* (Pers.) Rostr. under Artificial Inoculation. Indian J. Mycol. Plant Pathol..

[20] Ilyass M.B., Ahmad M.I., Bajwa M.A. (1999). Evaluation of Inoculation Methods and Screening of Wheat Germplasm against Loose Smut of Wheat. Pak. J. Agric. Sci..

[21] R Core Team (2017). R: A Language and Environment for Statistical Computing 4.1.3.

[22] Mourad A.M.I., Sallam A., Belamkar V., Wegulo S., Bowden R., Jin Y., Mahdy E., Bakheit B., El-Wafaa A.A., Poland J. (2018). Genome-Wide Association Study for Identification and Validation of Novel SNP Markers for Sr6 Stem Rust Resistance Gene in Bread Wheat. Front. Plant Sci..

[23] Poland J.A., Rife T.W. (2012). Genotyping-by-Sequencing for Plant Breeding and Genetics. Plant Genome.

[24] Bradbury P.J., Zhang Z., Kroon D.E., Casstevens T.M., Ramdoss Y., Buckler E.S. (2007). TASSEL: Software for Association Mapping of Complex Traits in Diverse Samples. Bioinformatics.

[25] Liu K., Muse S.V. (2005). PowerMarker: An Integrated Analysis Environment for Genetic Marker Analysis. Bioinformatics.

[26] Shin J.-H., Blay S., McNeney B., Graham J. (2006). LDheatmap: An R Function for Graphical Display of Pairwise Linkage Disequilibria between Single Nucleotide Polymorphisms. J. Stat. Softw..

[27] Dray S., Dufour A.B. (2007). The Ade4 Package: Implementing the Duality Diagram for Ecologists. J. Stat. Softw..

[28] Dhitaphichit P., Jones P., Keane E.M. (1989). Nuclear and Cytoplasmic Gene Control of Resistance to Loose Smut (*Ustilago tritici* (Pers.) Rostr.) in Wheat (*Triticum aestivum* L.). Theor. Appl. Genet..

[29] Duan R., Xiong H., Wang A., Chen G. (2015). Molecular Mechanisms Underlying Hull-Caryopsis Adhesion/Separation Revealed by Comparative Transcriptomic Analysis of Covered/Naked Barley (*Hordeum vulgare* L.). Int. J. Mol. Sci..

[30] Kumar J., Pratap A., Solanki R.K., Gupta D.S., Goyal A., Chaturvedi S.K., Nadarajan N., Kumar S. (2012). Genomic Resources for Improving Food Legume Crops. J. Agric. Sci..

[31] Alqudah A.M., Sallam A., Baenziger P.S., Börner A. (2020). GWAS: Fast-Forwarding Gene Identification and Characterization in Temperate Cereals: Lessons from Barley—A Review. J. Adv. Res..

[32] Liu B.H. (1998). Statistical Genomics: Linkage, Mapping, and QTL Analysis.

[33] Moursi Y.S., Thabet S.G., Amro A., Dawood M.F.A., Stephen Baenziger P., Sallam A. (2020). Detailed Genetic Analysis for Identifying QTLs Associated with Drought Tolerance at Seed Germination and Seedling Stages in Barley. Plants.

[34] Dawood M.F.A., Moursi Y.S., Amro A., Baenziger P.S., Sallam A. (2020). Investigation of Heat-Induced Changes in the Grain Yield and Grains Metabolites, with Molecular Insights on the Candidate Genes in Barley. Agronomy.

[35] Mourad A.M.I., Sallam A., Belamkar V., Wegulo S., Bai G., Mahdy E., Bakheit B., El-Wafa A.A., Jin Y., Baenziger P.S. (2019). Molecular Marker Dissection of Stem Rust Resistance in Nebraska Bread Wheat Germplasm. Sci. Rep..

[36] Abou-Zeid M.A., Mourad A.M.I. (2021). Genomic Regions Associated with Stripe Rust Resistance against the Egyptian Race Revealed by Genome-Wide Association Study. BMC Plant Biol..

[37] Mourad A.M.I., Sallam A., Belamkar V., Mahdy E., Bakheit B., El-wafaa A.A., Baenziger P.S. (2018). Genetic Architecture of Common Bunt Resistance in Winter Wheat Using Genome- Wide Association Study. BMC Plant Biol..

[38] Raffaele S., Leger A., Roby D. (2009). Very Long Chain Fatty Acid and Lipid Signaling in the Response of Plants to Pathogens. Plant Signal. Behav..

[39] Ma L., Li G. (2018). FAR1-Related Sequence (FRS) and FRS-Related Factor (FRF) Family Proteins in Arabidopsis Growth and Development. Front. Plant Sci..

[40] Wang X., Kong L., Zhi P., Chang C. (2020). Update on Cuticular Wax Biosynthesis and Its Roles in Plant Disease Resistance. Int. J. Mol. Sci..

[41] Cao A., Xing L., Wang X., Yang X., Wang W., Sun Y., Qian C., Ni J., Chen Y., Liu D. (2011). Serine/Threonine Kinase Gene Stpk-V, a Key Member of Powdery Mildew Resistance Gene Pm21, Confers Powdery Mildew Resistance in Wheat. Proc. Natl. Acad. Sci. USA.

[42] Brueggeman R., Drader T., Kleinhofs A. (2006). The Barley Serine/Threonine Kinase Gene Rpg1 Providing Resistance to Stem Rust Belongs to a Gene Family with Five Other Members Encoding Kinase Domains. Theor. Appl. Genet..

[43] Fu D., Uauy C., Distelfeld A., Blechl A., Epstein L., Chen X., Sela H., Fahima T., Dubcovsky J. (2009). A Kinase-START Gene Confers Temperature-Dependent Resistance to Wheat Stripe Rust. Science.

[44] Kobe B., Kemp B.E., Bradshaw R., Dennis E. (2003). Principles of Kinase Regulation. Handbook of Cell Signaling.

[45] Thomas P.L., Metcalfe D.R. (1984). Loose Smt Resistance in Two Introduction of Barely from Ethiopia. Can. J. Plant Sci..

[46] Bertan I., De Carvalho F.I.F., Oliveira A.C. (2007). De Parental Selection Strategies in Plant Breeding Programs. J. Crop Sci. Biotechnol..

