# Supplementary material for: Identification of Putative SNP Markers Associated with Resistance to Egyptian Loose Smut Race(s) in Spring Barley
Source: Genes (Basel). 2022 Jun 16;13(6):1075. doi: 10.3390/genes13061075 (PMC9223236; doi:10.3390/genes13061075)
Supplement: Supplementary file 1 [file genes-13-01075-s001.zip › Supplementary figures_ab.pptx]

## Slide 1
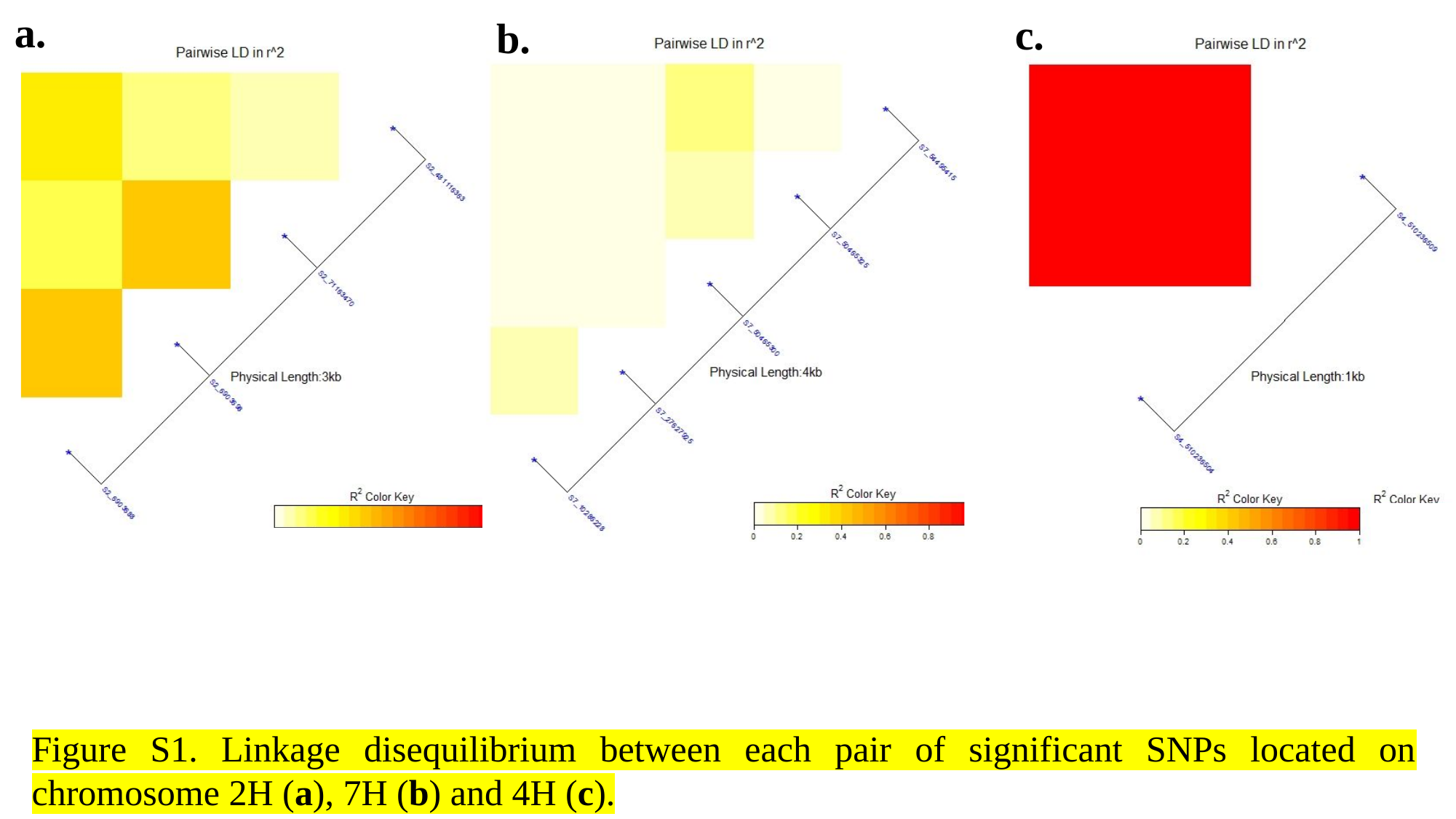

a.
c.
b.
Figure S1. Linkage disequilibrium between each pair of significant SNPs located on chromosome 2H (a), 7H (b) and 4H (c).
